# LINC01939 inhibits the metastasis of gastric cancer by acting as a molecular sponge of miR-17-5p to regulate EGR2 expression

**DOI:** 10.1038/s41419-019-1344-4

**Published:** 2019-01-25

**Authors:** Mi Chen, Li Fan, Si-Min Zhang, Yong Li, Peng Chen, Xin Peng, Dong-Bo Liu, Charlie Ma, Wen-Jie Zhang, Zhen-Wei Zou, Pin-Dong Li

**Affiliations:** 1 Cancer Center, Union Hospital, Tongji Medical College, Huazhong University of Science and Technology, Wuhan, 430022 China; 2 Department of Emergency, Wuhan Children’s Hospital (Wuhan Maternal and Child Healthcare Hospital), Tongji Medical College, Huazhong University of Science and Technology, Wuhan, 430022 China; 3 Department of Oncology, Renmin Hospital, Hubei University of Medicine, Shiyan, 442000 China; 4 Cancer Center, Tongji Hospital, Tongji Medical College, Huazhong University of Science and Technology, Wuhan, 430030 China; 5 Department of Radiation Oncology, Fox Chase Cancer Center, Philadelphia, PA 19111 USA; 6 Department of Pathology, Shihezi University School of Medicine, Shihezi, Xinjiang 832002 China

## Abstract

Accumulating evidence have suggested that long noncoding RNAs (lncRNAs) are known to regulate diverse tumorigenic processes. Recently, a novel lncRNA LINC01939 was underexpressed and emerged as a tumor suppressive lncRNA in gastric cancer (GC). In this study, we aimed to investigate the biological function and molecular mechanism of LINC01939 in GC. We found that LINC01939 expression was significantly downregulated in GC tissues and cell lines. Low expression of LINC01939 was correlated with tumor metastasis and shorter survival in GC patients. Functionally, LINC01939 overexpression remarkably inhibited the invasion and migration of GC cells in vitro and in vivo. Mechanistically, LINC01939 regulated the expression of early growth response 2 (EGR2) protein by competitively binding to miR-17-5p. Upregulation of miR-17-5p reversed GC metastasis and EMT process caused by LINC01939 by rescue analysis. Taken together, these results suggested that LINC01939 repressed GC invasion and migration by functioning as a ceRNA for miR-17-5p to regulate EGR2 expression. Our findings provided a novel prognostic marker and therapeutic target for GC patients.

## Introduction

Among the gastrointestinal malignances, gastric cancer (GC) is the most common cancer worldwide, and it mainly occurs in Eastern Asia including China and Japan^1^. A recent study showed that GC ranks as the second highest incidence rate and mortality rate among all cancer in China^2^. Currently, the primary treatments for advanced GC are surgery, chemotherapy and radiotherapy^3^. However, the 5-year survival rate of advanced GC patients after treatment is still unsatisfactory because of the high rate of metastasis^4^. Therefore, exploration of the molecular mechanism underlying GC metastasis and identification of novel biomarkers for predicting GC metastasis is urgently needed.

In mammals, it is estimated that up to 90% of the genomic DNA is transcribed with only 2% translated into proteins^5^. The majority of transcribed DNA encode a multitude of short and long noncoding RNAs (ncRNAs) which are classified as microRNAs (miRNAs), long noncoding RNAs (lncRNAs), circular RNAs and pseudogenes^6^. LncRNAs were previously regarded as “junk” or transcriptional noise owing to lack of protein-coding capacity, but more and more emerging evidences have demonstrated that lncRNAs exhibit complicated functions in gene transcription and protein regulation^7,8^. As expected, lncRNAs are considered as a new class of indispensable regulators involved in the progression and metastasis of cancer^9,10^. In gastric cancer, upregulation of lncRNA HOTAIR, MALAT1 and Linc00152 promoted cancer migration and invasion via several mechanisms including competitive endogenous RNA (ceRNA), epigenetic modification, transcription regulation, et al^11–13^. Hence, lncRNAs serve as new biomarkers for metastatic prediction and therapeutic targets for metastasis blocking in GC.

A recent study reported that LINC01939 was underexpressed and associated with clinical stage and lymphatic metastasis of GC patients^14^. However, the biological functions and underlying mechanisms of LINC01939 in GC is poorly understood. In this study, we found that LINC01939 expression was significantly reduced in GC tissues and cell lines. Low expression of LINC01939 was positively associated with GC metastasis and poor survival of GC patients. We further revealed that LINC01939 inhibited GC metastasis and EMT processes by acting as a molecular sponge or a ceRNA for miR-17-5p. Moreover, overexpression of LINC01939 exerted its tumor-suppressive effect through increasing the expression of early growth response 2 (EGR2) protein by sponging miR-17-5p. Our results also demonstrated that LINC01939/miR-17-5p/EGR2 axis regulates GC metastasis by inhibiting EMT pathway, which may shed light on their targeted applications in GC metastasis.

## Results

### Reduced expression of LINC01939 in GC tissues and the predictive value of LINC01939 in GC patients

**Fig. 1:**
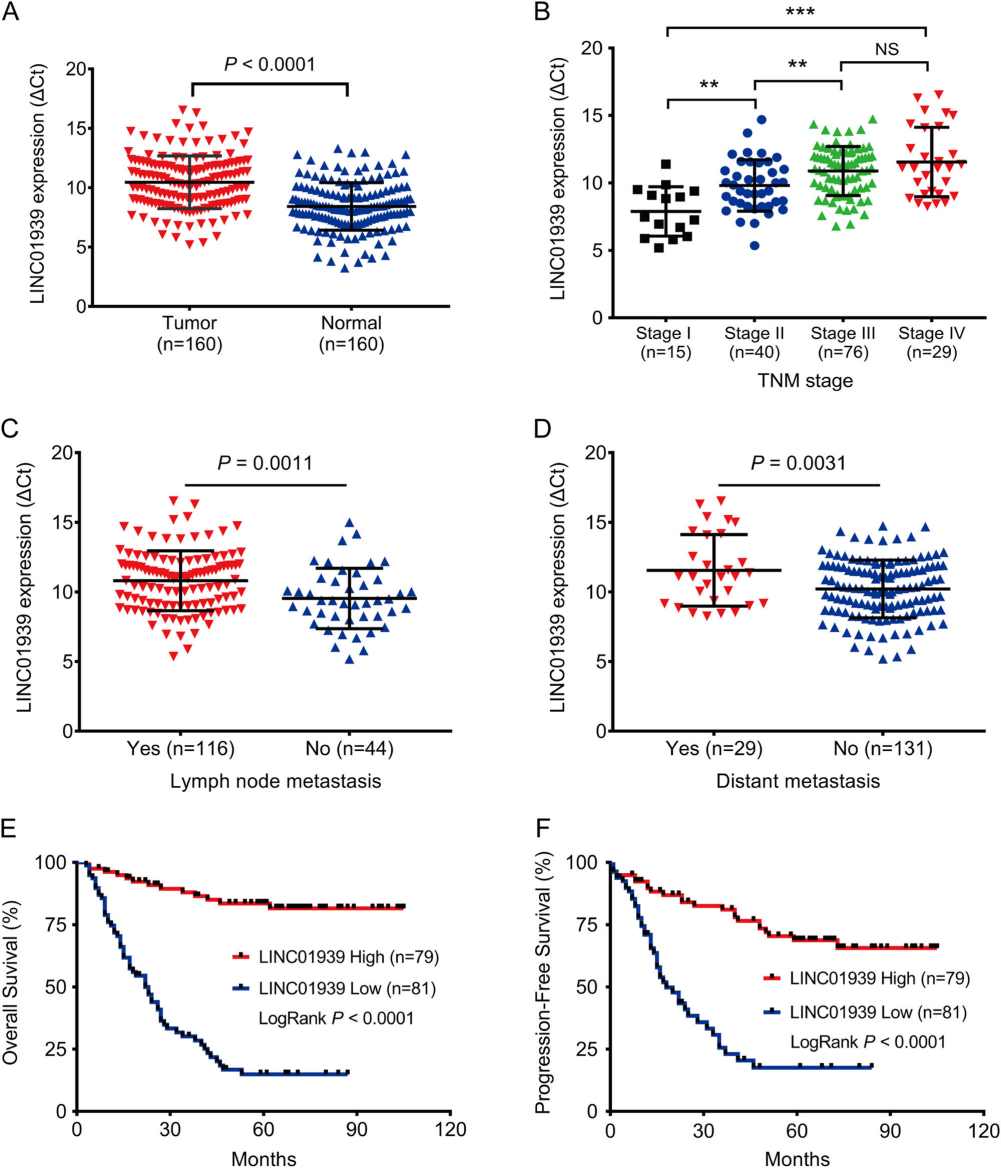
LINC01939 is significantly down-regulated and associated with adverse survival of GC patients. **a** Relative expression of LINC01939 in 160 paired gastric cancer (GC) tissues and matched normal tissues by RT-PCR. Results were presented as Δcycle threshold (ΔCt) in tumor tissues relative to normal tissues. **b** Relative expression of LINC01939 in different clinical stages. LINC01939 expression was lower in patients with advanced-stage GC than those with early-stage GC (^**^P < 0.01; ^***^P < 0.005, NS, no significant). The horizontal lines and vertical error bars show mean ± SD (**c**) and (**d**) Relative expression of LINC01939 in GC patients with/without lymph node metastasis or with/without distant metastasis. **e** and **f** Kaplan-Meier plots of overall-survival (OS) and progression-free survival (PFS) in GC patients with high (*n* = 79) and low (*n* = 81) levels of LINC01939. The OS and PFS was significantly shorter in patients with LINC01939 high expression than those with low expression (all *P* < 0.0001)

To assess the correlation between LINC01939 and GC metastasis, we performed reverse transcription and quantitative PCR (RT-PCR) to investigate the expression of LINC01939 in a larger cohort of GC tissues. The result showed that LINC01939 expression was significantly reduced in tumor tissues compared with matched normal tissues (*P* < 0.001, Fig. 1a). Further analysis indicated that advanced TNM stage, lymph node metastasis and distance metastasis were negatively correlated with the expression of LINC01939 (Fig. 1b–d), suggesting that LINC01939 may inhibit GC progression, particularly metastasis. These results were in concordance with previous findings in GC^14^. The patient cohort was then divided into low and high LINC01939 expression groups based on the median expression level (median, 0.20) as the cut-off value. The correlation between LINC01939 expression and clinicopathological parameters by Chi-square test was displayed in Supplementary Table S1.

Since LINC01939 is correlated with GC metastasis, we hypothesized that low expression of LINC01939 in GC patients indicated poor prognosis. To corroborate this hypothesis, we analyzed overall survival (OS) and progression-free survival (PFS) of GC patients with low or high LINC01939 expression by Kaplan–Meier method and Log-rank test. As expected, the 5-year OS and PFS of GC patients with high LINC01939 expression were 83.5% and 68.7% respectively, which were significantly better than those with low LINC01939 expression (14.8% and 17.5% respectively) (all *P* < 0.001, Fig. 1e, f). Subsequently, univariate and multivariate analyses were conducted on LINC01939 and other clinicopathological parameters to determine the prognostic significance of LINC01939. Univariate analysis revealed that LINC01939 expression, TNM stage, lymph node metastasis and distant metastasis were significant prognostic factors for OS and PFS (all *P* < 0.01, Table 1). But peritoneum dissemination was only significant predictor for OS in GC patients. Furthermore, multivariate Cox regression analysis demonstrated that LINC01939 is an independent survival predictor for OS and PFS in GC patients (all *P* < 0.001, Table 1). In addition, TNM stage and distant metastasis were also independent risk predictors for OS and PFS. These results suggest that LINC01939 may serve as a potential biomarker for predicting GC progression.

**Table 1:** Univariate and multivariate analysis of LINC01939 associated with OS and PFS in GC patients

Characteristics	OS			PFS		
	Univariate *P* value	Multivariate analysis		Univariate *P* value	Multivariate analysis	
		*P* value	HR (95% CI)		*P* value	HR (95% CI)
Sex (Male vs. Female)	0.289			0.481		
Age. years (≥60 vs. <60	0.495			0.206		
Tumor size (≥5 cm vs. <5 cm)	0.175			0.563		
Differentiation (Poor vs. Moderate/Well)	0.289			0.552		
TNM stage (III-IV vs. II-I)	<0.001	0.437	1.388 (0.607–3.169)	<0.001	0.038	2.701 (1.056–6.909)
Lymph node metastasis (Yes vs. No)	0.004	0.372	1.481 (0.625–3.510)	0.006	0.609	0.786 (1.312–1.978)
Distant metastasis (Yes vs. No)	<0.001	0.006	3.280 (1.394–7.716)	0.002	0.041	1.921 (1.026–3.598)
Peritoneum dissemination (Yes vs. No)	0.005	0.521	0.722 (0.267–1.954)	0.387		
LINC01939 expression (high vs. low)	<0.001	<0.001	0.112 (0.059–0.210)	<0.001	<0.001	0.253 (0.147–0.435)

*TNM stage* tumor-node-metastasis stage, *OS* overall survival, *PFS* progression-free survival, *HR* hazard ratio, *CI* confidence interval

### LINC01939 inhibits GC invasion and migration in vitro and in vivo

**Fig. 2:**
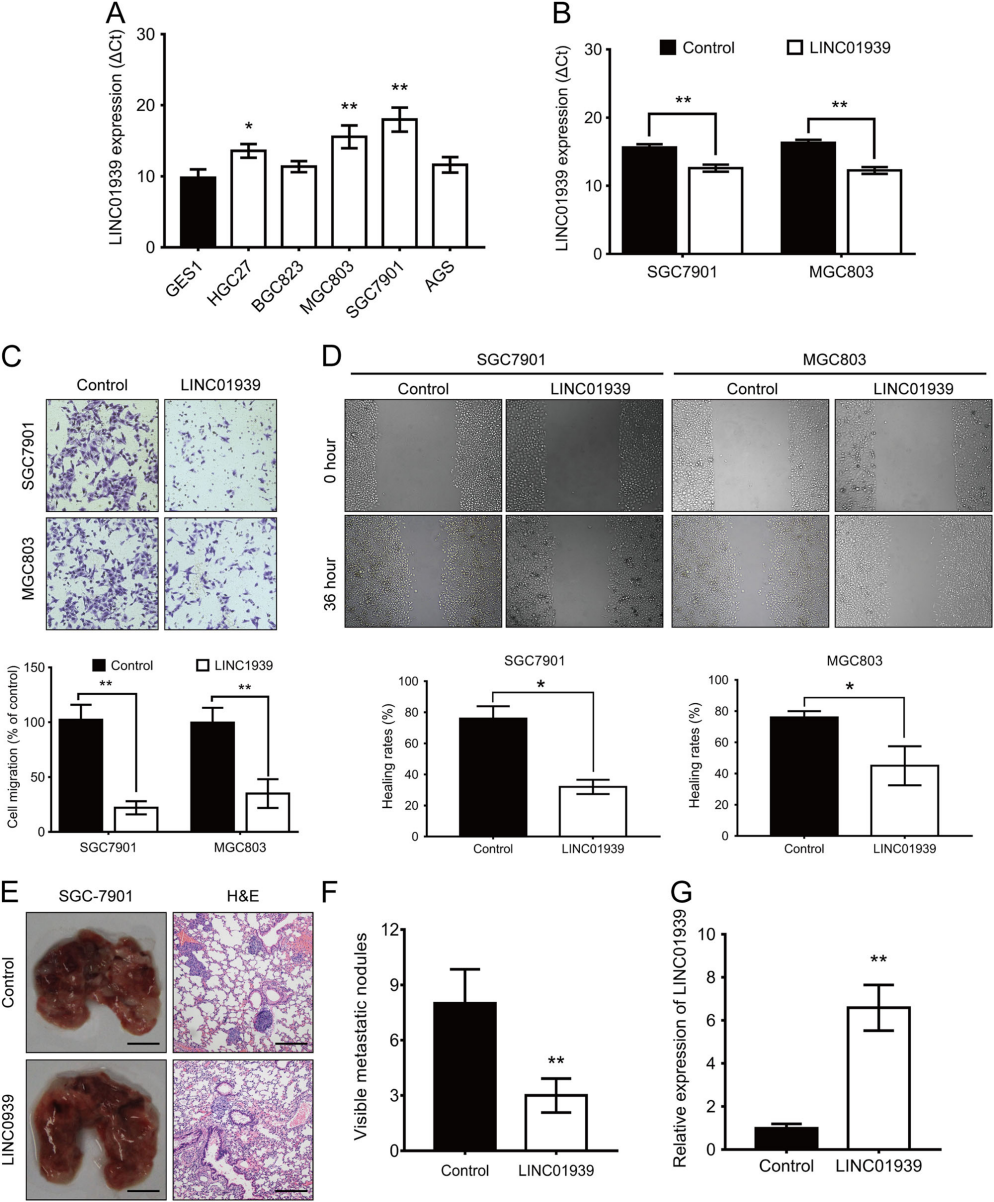
LINC01939 inhibits the invasion and metastasis in GC cells in vitro and in vivo. **a** Relative expression of LINC01939 in 5 gastric cancer cell lines compared with that of the normal gastric epithelial cell line GES1. **b** Relative expression of LINC01939 in SGC7901 and MGC803 cells after transfection with an expression vector pCMV-LINC01939 by RT-PCR. **c** Migration assays of SGC7901 and MGC803 cells with LINC01939 overexpression by transwell assays (quantification in bottom histogram). **d** Wound healing assays of SGC7901 and MGC803 cells with LINC01939 overexpression. Quantifications were shown in bottom histogram. **e** Representative images (left panel) and HE staining (right panel) of pulmonary metastatic nodes in mice injected with SCG7901-LINC01939 cells compared with those injected with SGC7901 NC cells. Scale bar, left: 5 mm; right: 100 μm. **f** The mean number of pulmonary metastatic nodes were 8.6 and 3.3 for SGC7901-control and SGC7901-LINC01939 groups, respectively. **g** Relative expression of LINC01939 gene in pulmonary metastatic nodes in above two groups confirmed by RT-PCR. Error bars: mean ± SD. ^*^ *P* < 0.05; ^**^P < 0.01

Before conducting the function experiments of LINC01939, we predicted the coding capacity of LINC01939 by online tool CPAT. The result displayed that LINC01939 had no protein-coding capacity (Supplementary Figure S1A). According to the correlation between LINC01939 expression and GC metastatic factors, we focused on the biological functions of LINC01939 in GC metastasis. We first measured the expression of LINC01939 in some common GC cells. The results showed that LINC01939 was significantly down-regulated in HGC27, BGC823, MGC803, SGC7901 and AGS cells (Fig. 2a). For confirming our results, we performed RT-PCR to detect the relative expression of LINC01939 by another specific primers of LINC01939 in GC tissues and cell lines. These results were consistent with abovementioned findings (Supplementary Figure S1B and S1C). SGC7901 and MGC803 cells whose LINC01939 expression were the lowest in the detected GC cell lines, were selected to study the biological function of LINC01939. An expression vector pCMV-LINC01939 was transfected into SGC7901 and MGC803 cells and the efficiency of LINC01939 overexpression was confirmed by RT-PCR (Fig. 2b). Transwell assay showed that LINC01939 overexpression significantly decreased the potential of invasion in SGC7901 and MGC803 cells (Fig. 2c). Meanwhile, overexpression of LINC01939 led to significant attenuates in the capacity of migration by wound healing assay (Fig. 2d). Therefore, our data suggest that LINC01939 overexpression could inhibit GC invasion and migration in vitro.

To further investigate the in vivo effect of LINC01939 on lung metastasis, SGC7901-control and SGC7901-LINC01939 cells were injected into the tail vein of nude mice. The numbers of pulmonary metastatic nodes were significantly reduced in mice injected with SGC7901-LINC01939 cells compared with the numbers in those injected with SGC7901-control cells (Fig. 2e, f). RT-PCR assay confirmed that overexpression of LINC01939 remarkably increased LINC01939 expression in metastatic nodes in the lung (Fig. 2g). Altogether, LINC01939 overexpression suppresses GC metastasis in vivo.

### LINC01939 inhibits miR-17-5p through directly binding

**Fig. 3:**
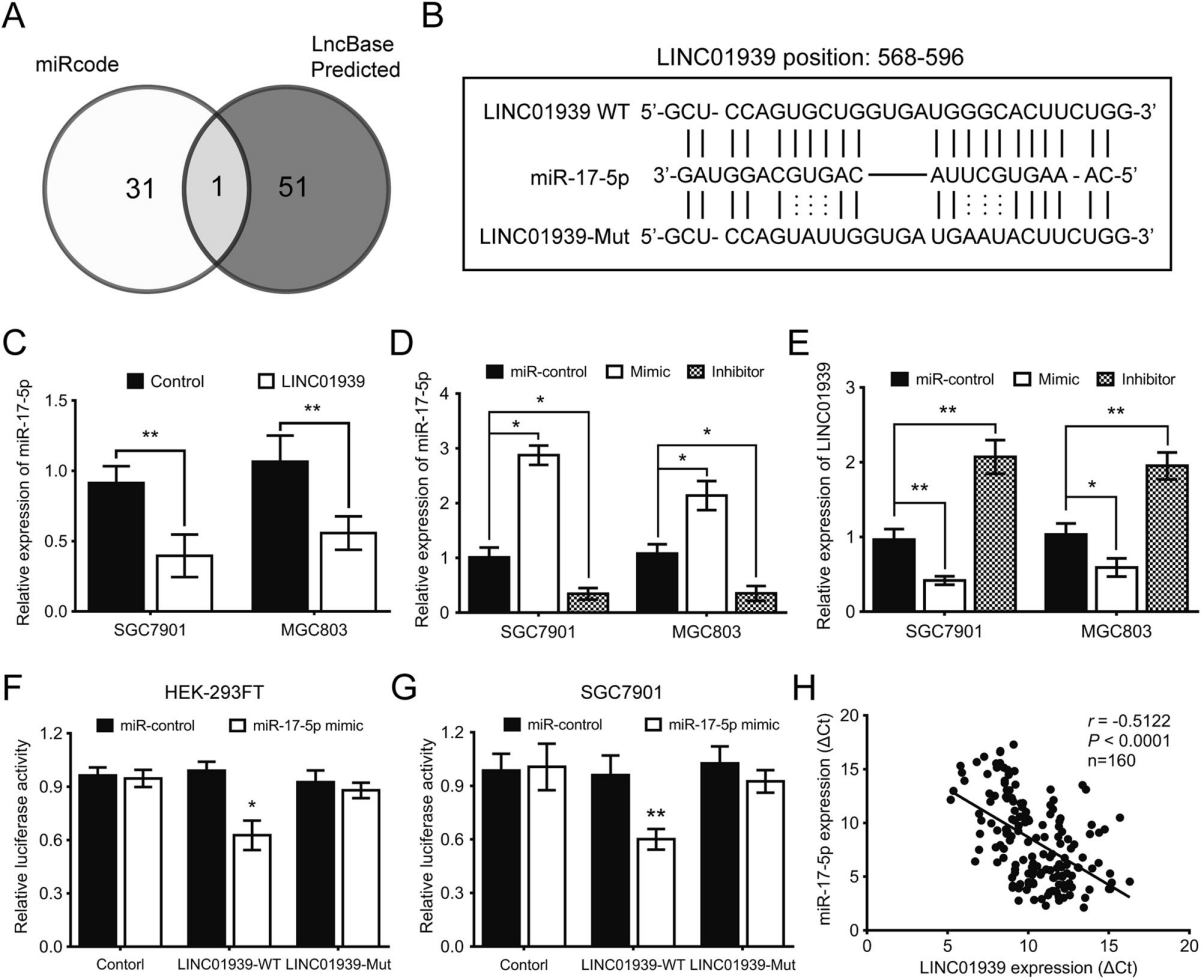
LINC01939 is negative regulated by miR-17-5p in gastric cancer. **a** Venn diagrams showing the number of potential miRNAs targeting LINC01939, as predicted by two databases: miRcode and LncBase Predicted. **b** Predicted binding sites of miR-17-5p and LINC01939. **c** Overexpression of LINC01939 leads to decrease miR-17-5p expression in SGC7901 and MGC803 cells. **d** RT-PCR analysis confirmed the miR-17-5p ectopic expression and miR-17-5p inhibition in SGC7901 and MGC803 cells. **e** Expression of LINC01939 after transfection of miR-17-5p mimic or inhibitor in SGC7901 and MGC803 cells. **f** and **g** Luciferase reporter assay in human embryonic kidney 293FT (HEK-293FT) or SGC7901 cells co-transfected with wild type (WT) or mutated (Mut) LINC01939 reporter and miR-17-5p mimic. **h** Correlation between LINC01939 and miR-17-5p expression in 160 GC tissues. Means ± SD (*n* = 3) are shown in (**c**), (**d**), (**e**), (**f**) and (**g**). ^*^P < 0.05 and ^**^P < 0.01 versus control cells

Increasing evidences showed that cytoplasmic lncRNAs can function as sinks for pools of active miRNAs, functionally liberating mRNAs to mediate several biological processes^15^. RT-PCR was then performed to examine differential expression of LINC01939 in subcellular fractions of BGC823, AGS and GES1 cells. The results indicated that LINC01939 predominantly localized to the cytoplasm (Supplementary Figure S1D). Therefore, we hypothesized that LINC01939 functions as a ceRNA for certain miRNAs to regulate GC metastasis. The potential conjugated miRNAs were predicted using publicly available algorithms (miRcode and LncBase Predicted) and the result showed miR-17-5p was the predicted target of LINC01939 (Fig. 3a). The bioinformatic method predicts the alignment of the complementary binding of LINC01939 and miR-17-5p (Fig. 3b). Then we found that miR-17-5p level was significantly decreased in SGC7901 and MGC803 cells with LINC01939 overexpression (Fig. 3c). Subsequently, we investigated whether miR-17-5p also regulate LINC01939 expression. The results showed that ectopic expression of miR-17-5p significantly decreased LINC01939 expression, whereas miR-17-5p inhibition remarkably increased the expression of LINC01939 (Fig. 3d, e). Besides, to confirm the specific binding of the two RNAs, miR-215 and miR-422a were randomly selected for control, and RNAHybrid online program verified that LINC01939 will not bind with miR-215 or miR-442a. RT-PCR assay further demonstrated that overexpression of LINC01939 did not affect the expression of miR-215 or miR-442a in SGC7901 and MGC803 cells (Supplementary Figure S2A and S2B). We also found that miR-17-5p expression was significantly reduced in untreated GES1 cells compared with SGC7901 cells (Supplementary Figure S2C). As shown in Supplementary Figure S2D, transfection of GES1 cells with miR-17-5p mimic or inhibitor similarly decreased or increased the expression of LINC01939.

Further experiments were conducted to determine whether LINC01939 affect the processing of miR-17-5p. We firstly looked for whether LINC01939 could modulate the expression of pri-miR-17-29, the precursor of miR-17-5p. The results showed that overexpression of LINC01939 in SGC7901 and MGC803 cells did not affect the expression of pre-miR-17-29 (Supplementary Figure S3A). To determine whether LINC01939 can directly bind to miR-17-5p by competitively combining with a miRNA response element (MRE), we first constructed luciferase reporters, which contain wild-type (WT) or mutated (Mut) miR-17-5p binding sites (Fig. 3b). As shown in Fig. 3f, g, transfection of HEK-293FT and SGC7901 cells with miR-17-5p mimic significantly reduced the luciferase activities of the LINC01939-WT reporter vector but not control or LINC01939-Mut reporter vector, confirming the direct correlation between miR-17-5p and LINC01939. In addition, RT-PCR assay further verified that compared with matched normal tissues, miR-17-5p expression was increased in GC tissues (Supplementary Figure S3B). There was an inverse correlation between miR-17-5p and LINC01939 in 160 GC tissues (Fig. 3h). A similar phenomenon was observed in 160 corresponding “normal” gastric tissues (Supplementary Figure S3C) Collectively, our findings suggest that LINC01939 can suppress miR-17-5p expression via directly binding at the MRE.

### LINC01939 increases EGR2 expression acting as a ceRNA of miR-17-5p

**Fig. 4:**
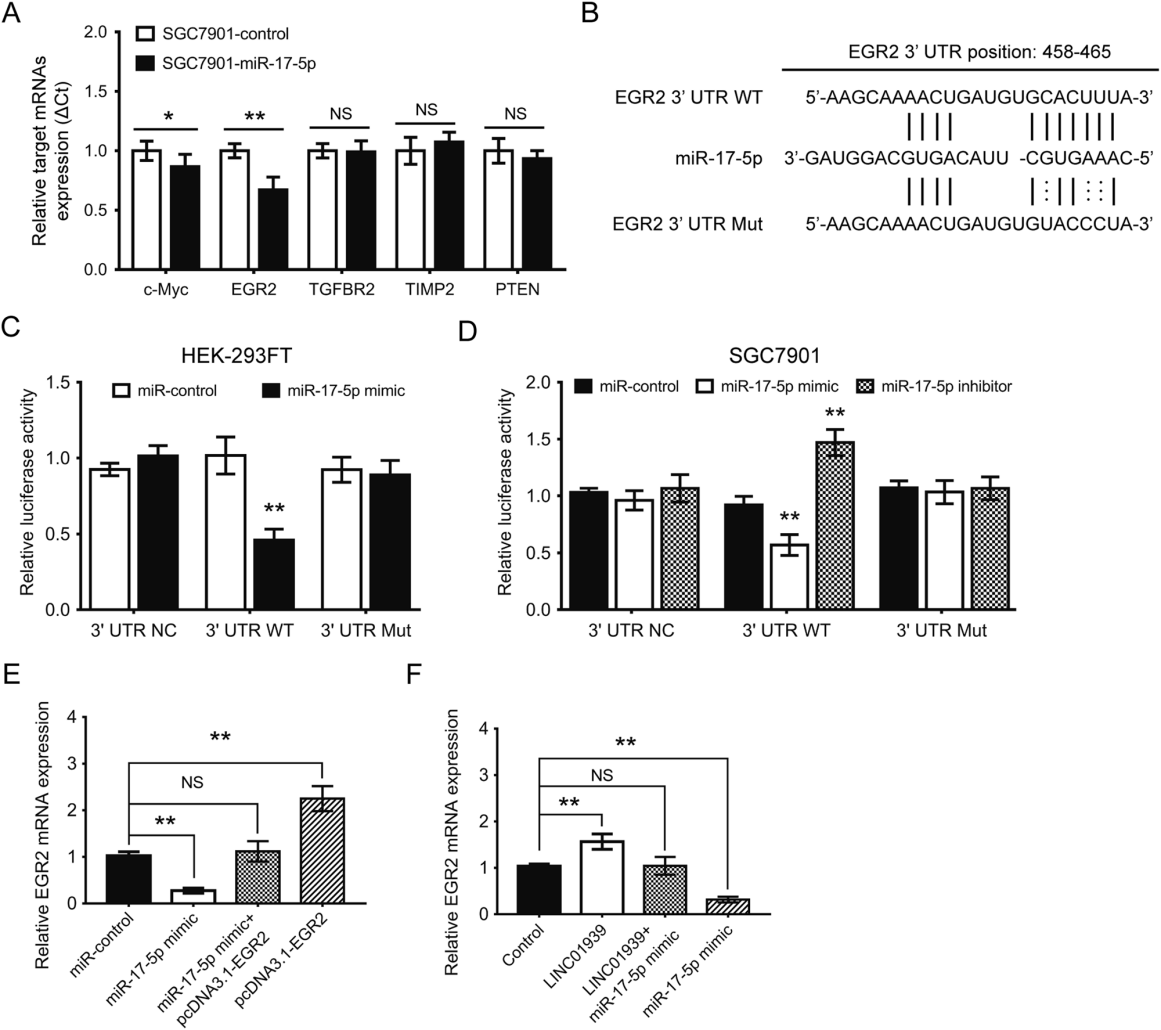
LINC01939 increases expression of miR-17-5p targeted gene EGR2 in GC. **a** The expression of five candidate mRNAs of miR-17-5p targeted genes confirmed by RT-PCR in SGC7901-control and SGC7901-miR-17-5p cells. **b** The potential miR-17-5p binding sites in EGR2 mRNA 3′-UTR as predicted by the Targetscan algorithm. Mutation (Mut) was introduced into EGR2 3′-UTR to disrupt base-pairing with miR-17-5p seed sequence. **c** and **d** Dual-luciferase assays in HEK-293FT and SGC7901 cells indicated a significant reduction or increment luciferase activities after co-transfection of miR-17-5p mimic or inhibitor and the wild-type (WT) EGR2 mRNA 3′-UTR, but not the mutant-type (Mut) EGR2 3′-UTR. **e** EGR2 mRNA level in SGC7901 cells following ectopic expression of miR-17-5p and/or EGR2 expression vector lacking the 3′UTR. **f** EGR2 mRNA level in SGC7901 cells following overexpression of LINC01939 and/or miR-17-5p mimic. Error bars: mean ± SD, *n* = 3. *NS* no significant), ^*^P < 0.05 and ^**^P < 0.01 versus corresponding control

It is generally accepted that miRNAs could bind to the 3′-untranslated regions (3′-UTR) of protein coding genes and thus mediate their expressions^8^. Based on a large mount of literature, regulative correlations of miR-17-5p and five metastasis-related target mRNAs (c-Myc, EGR2, TGFBR2, TIMP2 and PTEN) have been well documented in several human cancers^16–21^. The expression levels of above genes were further verified via RT-PCR. The results showed that early growth response 2 (EGR2) was consistently downregulated in SGC7901 or MGC803-miR-17-5p cells compared with matched SGC7901- or MGC803-control cells (*P* < 0.01, Fig. 4a and Supplementary Figure S3D). For confirming above results, we again performed RT-PCR to investigate the expression of EGR2 by another specific primers of EGR2 in GC tissues and cell lines. These results showed that EGR2 expression was significantly reduced in 30 GC tissues (Supplementary Figure S3E), and the expression level of EGR2 in the majority of 5 GC cell lines was lower than in the GES-1 (Supplementary Figure S3F). Using the Targetscan algorithms, a potential miR-17-5p binding site was identified in the EGR2 3′-UTR (Fig. 4b). We then introduced a mutation into EGR2 3′-UTR to disrupt base-pairing with the miR-17-5p seed sequence (Fig. 4b). As expected, luciferase reporter assays in 293FT cells revealed that miR-17-5p mimic significantly inhibited the EGR2 transcriptional expression in EGR2 3′-UTR wild type (WT). On the contrary, miR-17-5p mimic failed to influence its expression in EGR2 3′-UTR Mut (Fig. 4c). We also conducted a luciferase activity assay in SGC7901 cells and found the similar results (Fig. 4d).

According to the ceRNA concept, lncRNA functions as a ceRNA to exert its regulatory roles in cancer progression and metastasis^22^. We found that LINC01939 shares the same miR-17-5p binding sites with EGR2 mRNA 3′-UTR (Fig. 3b and Fig. 4b). To demonstrate that LINC01939 functions as a ceRNA in regulating EGR2 through competitively binding to miR-17-5p, we conducted RT-PCR to observe the expression of EGR2. As shown in Fig. 4e, the mRNA level of EGR2 was significantly attenuated by miR-17-5p ectopic expression, and this reduction was retrieved by co-transfected with pcDNA3.1-EGR2 vector. To our interest, upregulation of LINC01939 promoted EGR2 mRNA expression, which was significantly retracted by the overexpression of miR-17-5p (Fig. 4f). These data strongly indicated that LINC01939 regulates the expression of EGR2 mRNA in an miR-17-5p-dependent manner.

### LINC01939/miR-17-5p/EGR2 axis inhibits GC metastasis and EMT process

**Fig. 5:**
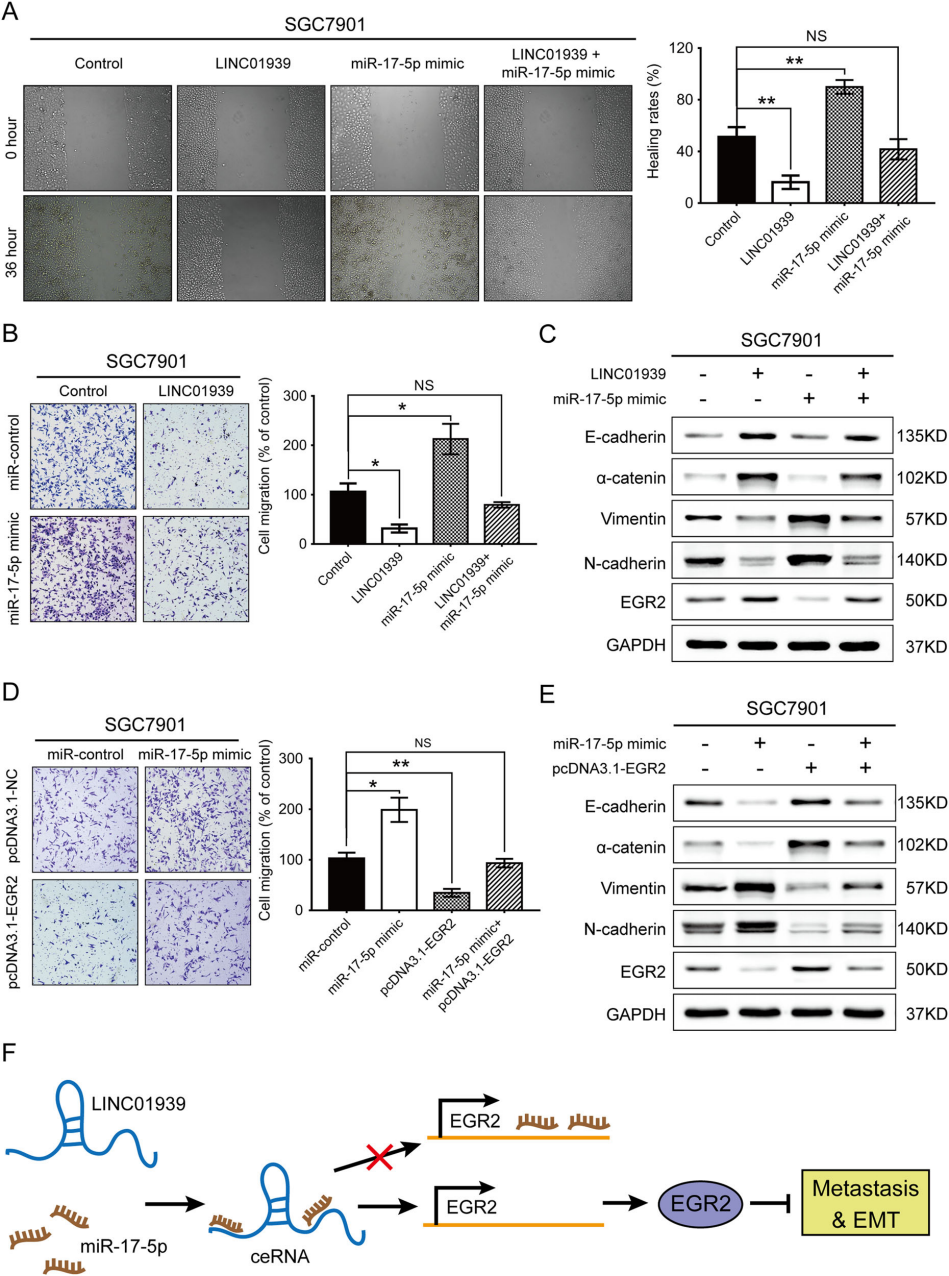
LINC01939 exerts anti-metastatic activities by inhibiting miR-17-5p and upregulating EGR2 expression. **a** Representative images of wound healing assay of SGC7901 cells with ectopic expression of LINC01939 and/or co-transfected with miR-17-5p mimic. Quantification was shown in right histogram. **b** Transwell assay of SGC7901 cells after overexpression of LINC01939 and/or co-transfected with miR-17-5p mimic. Representative images (left panel) and quantifications (right panel) were shown. Error bars: mean ± SD. NS, no significant, ^*^P < 0.05 and ^**^P < 0.01. **c** Western blots of EMT markers (E-cadherin, α-catenin, Vimentin and N-cadherin expression) and EGR2 protein in SGC7901 cells with LINC01939 overexpression and/or upregulation of miR-17-5p. **d** Transwell assay of SGC7901 cells after transfection with miR-17-5p mimic and/or pcDNA3.1-EGR2. Quantification was shown in right histogram. **e** Expression of EMT markers and EGR2 expression after upregulation of miR-17-5p and/or EGR2 genes in SGC7901 cells. **f** Schematic diagram of LINC01929 in GC metastasis. LINC01939 as a ceRNA sponges and inhibits miR-17-5p. The interaction of LINC01939 and miR-17-5p blocks or decreases the interaction of miR-17-5p and EGR2 mRNA 3′-UTR, then promotes the expression of EGR2 protein. The latter suppresses GC metastasis and EMT processes

To explore whether the anti-metastatic effect of LINC01939 in GC cells is miR-17-5p/EGR2 axis-dependent, we further investigated the effect of LINC01939 and/or miR-17-5p and/or EGR2 expression on migration and invasion through functional trials. Wound healing assay showed that cell migration was enhanced after transfection with miR-17-5p mimic (Fig. 5a), suggesting that the stimulating activity of miR-17-5p on tumor migration. As expected, the anti-metastatic effect of LINC01939 in SGC7901 cells could be rescued by co-transfected with miR-17-5p mimic (Fig. 5a). Similarly, LINC01939 could memorably attenuate the invasion ability stimulated by miR-17-5p mimic (Fig. 5b). EMT plays an important role in cancer metastasis^23^. Western blot assay revealed that LINC01939 overexpression led to increase the expression of the epithelial markers E-cadherin and α-catenin and decrease the expression of the mesenchymal markers vimentin and N-cadherin (Fig. 5c), indicating that LINC01939 may inhibit GC migration and invasion via regulating EMT pathway. Moreover, the inhibitory activity of LINC01939 on EMT pathway was restored by co-transfected with miR-17-5p mimic (Fig. 5c). In addition, we found that upregulation of miR-17-5p was able to enhance the invasion ability of SGC7901 cells, and the pro-invasive effect was contracted by co-transfected with a pcDNA3.1-EGR2 vector (Fig. 5d). Similarly, EGR2 overexpression could suppress the EMT process stimulated by miR-17-5p mimic in SGC7901 cells (Fig. 5e). Thus, our results suggest that LINC01939 may suppress GC metastasis and EMT process by targeting miR-17-5p to upregulate EGR2 expression.

## Discussion

In the present study, we found that reduced expression of LINC01939 is a common event in GC, indicating tumor suppressive role of LINC01939. However, the data from lnCAR database showed that LINC01939 expression is overexpressed in cholangiocarcinoma (CC)^14^, suggesting that different human cancer types might account for the different expression and function of LINC01939. In addition, we confirmed the positive association of low LINC01939 expression in GC tissues with GC metastasis and poor prognosis. Primed by these results, we suggest that LINC01939 has a cancer-specific expression pattern and can act as a potential biomarker to help identify patients at a higher risk of GC metastasis.

There has been wide consensus that dysregulation of lncRNAs are commonly investigated in gastrointestinal malignances including GC^3,24^. For example, lncRNA AGAP2-AS1 expression was highly upregulated in GC. AGAP2-AS1 promoted GC proliferation and metastasis by inhibiting CDKN1A and E-cadherin transcription^25^. Another interesting lncRNA AK023391 not only inhibited GC growth and invasion but also enhanced cell cycle and apoptosis by activating PI3K/Akt signaling pathway^26^. In the present study, LINC01939 also participated in GC metastasis and EMT processes in vitro and in vivo and was considered as a metastasis-associated lncRNA.

Further analysis found that the majority of LINC01939 located in cytoplasm. Emerging evidence demonstrated that cytoplasmic lncRNAs mainly function as ceRNAs or molecular sponges to competitively inhibit several miRNAs, then participate in the carcinogenesis of GC^15^. It has been reported that lncRNA LINC01133 acts as a ceRNA in regulating APC/Wnt/β-cantenin signaling pathway through competitively binding to miR-106a-3p in gastric cancer^22^. LncRNA MT1JP inhibited GC growth and metastasis by acting as a molecular sponge of miR-92a-3p to modulate FBXW7 expression^27^. Therefore, we speculated that LINC01939 may be a ceRNA in GC metastasis. Bioinformatic analysis and luciferase reporter assay confirmed that LINC01939 directly binds to miR-17-5p and functions as a sponge of miR-17-5p to upregulate the expression of EGR2 protein. It has been well-established that miR-17-5p was found to be overexpressed and to promote tumor growth and autophagy in many human cancers^28^. In gastric cancer, miR-17-5p was significantly upregulated in GC tissues and increased GC growth by repressing SOCS6 or modulating p21 and TP53INP1^29,30^. And miR-17-5p was also found to promote GC proliferation and migration via negatively regulating TGFBR2 expression^20^. In the present study, we again observed that miR-17-5p was overexpressed and promoted tumor invasion and migration in GC. What′s more, we first confirmed that overexpression of LINC01939 could reduce miR-17-5p expression, and LINC01939 suppressed GC metastasis and EMT which was restored by miR-17-5p mimic. These data suggest that LINC01939 exerts its anti-metastatic activities at least in part by regulating miR-17-5p expression.

Actually, our further study confirmed that LINC01939 and miR-17-5p showed heterogeneous expression in several human normal tissues (including GC) from online databases (data not shown). However, we could not compare their expression due to the feeble expression of the two RNAs in normal gastric tissues from healthy people. In addition, by virtue of artificial overexpression of LINC01939 and two potential miR-17-5p binding sites in LINC01939 gene, the molar concentration between the two RNAs in our study was about 1.5~ 2:1, which was consistent with our results in Fig. 3c–e.

Early growth response 2 (EGR2) is a transcription factor with three tandem C2H2-type zine fingers^31^. Previous studies have demonstrated that EGR2 is implicated in cell proliferation and cell cycle by regulating P53-induced apoptosis^32^. As a tumor suppressor gene, EGR2 expression was reduced in GC tissues and regulated by several miRNAs (miR-20a, miR-150 and miR-17-5p)^17,33,34^. Our study provided evidence to support the hypothesis that EGR2 is suppressed by miR-17-5p and that inhibition of EGR2 expression enhances GC metastasis, which was consistent with a previous study^17^. However, we could not clarify which downstream target genes of EGR2 are directly transactivated or transrepressed in regulating GC metastasis. Therefore, future studies to answer these questions are required.

## Conclusions

In summary, we determined that LINC01939 expression was significantly decreased in GC tissues and cell lines and predicted adverse outcomes for GC patients. Functional experiments showed that LINC01939 inhibited GC cell metastasis in vitro and in vivo. Mechanistically, we demonstrated that LINC01939 plays a ceRNA role in regulating EGR2 expression by competitively binding to miR-17-5p. LINC01939 suppresses GC metastasis and EMT process via upregulating EGR2 expression (Fig. 5f). Our study provided new sight into the post-transcriptional regulation mechanism of LINC01939 implicated in GC metastasis. LINC01939 may serve as a potential novel prognostic biomarker and therapeutic target for GC treatment.

## Materials and methods

### Gastric cancer samples collection and cell lines culture

We obtained 160 pairs of GC samples from the surgical resection tissues of GC patients of Cancer Center, Union Hospital, Tongji Medical College, Huazhong University of Science and Technology (Wuhan, China) between February 2007 and December 2011. None of our patients received any radiotherapy and/or chemotherapy before surgery. Clinical and pathological tumor staging was conducted according to the 7^th^ edition of the TNM classification of American Joint Committee on Cancer (AJCC). All the enrolled patients were notified of this research purpose and signed informed consent. This study was approved by the Institutional Review Board and Human Ethics Committee of Cancer Center, Union Hospital, Tongji Medical College, Huazhong University of Science and Technology.

GC cell lines were purchased from the Cell Bank of the Chinese Academy of Sciences (Shanghai, China), including HGC27, BGC823, MGC803, SGC7901 and AGS and the normal gastric epithelial cell line GES1. Human embryonic kidney (HEK) 293 FT cells was obtained from the American Type Culture Collection (ATCC). All GC cell lines were cultured in RPMI 1640 (Gibco) with 10% Fetal Bovine Serum (FBS, Gibco). HEK-293 FT cells was cultured in DMEM (Gibco) with 10% FBS. All cultures were tested for the presence of mycoplasma and cultured in a humidified 37 °C culture incubator in the presence of 5% CO_2_ and 20% O_2_.

### RNA extraction and Real-time PCR analysis (RT-PCR)

Total RNA from GC tissues or cells were isolated using TRizol reagent (Invitrogen, Carlsbad, CA, USA) according to the manufacturer’s instructions. 1 μg of total RNA was converted to cDNA by GoScript^TM^ Reverse Transcription System kit (Promega, Madison, USA) with oligo(dT) and random primers. Real-time PCR was conducted using GoTaq® qPCR Master Mix (Promega) on a Light Cycler 480 instrument (Roche, USA). The relative quantification of target genes was calculated using the comparative 2^-ΔΔCT^ method and normalized to GAPDH expression. For miRNA detection, cDNA was synthesized with All-in-one^TM^ First-Strand cDNA Synthesis Kit (GeneCopoeia, Rockville, USA). Quantitative detection of miR-17-5p and U6 with All-in-one^TM^ miRNA qRT-PCR Detection Kit (GeneCopoeia, Rockville, USA) was performed on ABI 7500 fast real-time PCR system (Applied Biosystems, Darmstadt, Germany) according to a standard method as described previously^35^. All samples were amplified in triplicate and small nuclear RNA(U6) was detected as an internal control. The primer sequences of target genes used in this study were shown in Supplementary Table S2. Special announcement: There are three transcripts of LINC01939 according to LNCipedia database. Our study focused on the important role of the longest transcript of LINC01939 (ENST00000445279) and designed the specific primers of this form.

### LncRNA coding capacity prediction

The coding capacity of LINC01939 was evaluated by Coding Potential Assessment Tool (CAPT, http://lilab.research.bcm.edu/cpat/). CPAT performs a logistic regression model built with four sequence features: open reading frame coverage, open reading frame size, hexamer usage bias and Fickett TESTCODE statistic. The cutoff value as human coding probability (CP) is set at 0.364. CP < 0.364 is considered as noncoding transcripts, whereas CP ≥ 0.364 is defined as coding transcripts^36^.

### Isolation of nuclear-cytoplasmic RNA

Nuclear and cytoplasmic RNA were separated and purified by Cytoplasmic & Nuclear RNA Purification Kit (Norgen Biotek, Canada) according to the manufacturer’s instructions. β-actin was detected as a cytoplasmic control and U6 was a nuclear control.

### Lentivirus packaging and infection

LINC01939 cDNA was cloned into the mammalian expression vector pLenti-GIII-CMV-Puro vector from Applied Biological Materials (ABM Inc., BC, Canada). The viral supernatants were added into SGC7901 and MGC803 cells to construct stable LINC01939 overexpression cell lines. Cells were further treated with puromycin (2 μg/ml) for one weeks to select stably transfected cells. The miR-17-5p inhibitor and mimic were purchased from GenePharma (Shanghai, China). SGC7901 and MGC803 cells were transfected with Lipofectamine3000 (Life Technologies Corporation, Carlsbad, CA, USA). 48 h after transfection, cells were collected and used for subsequent experiments.

### Transwell assay and wound healing assay

GC cells were suspended in 200 ul serum-free medium and added to the upper chamber with 8-μm pores (Corning Costar, NY, USA), while 600 ul medium containing 20% FBS was placed in the lower chamber as chemoattractant. After incubation for 24 h, cells in the filter were immersed in methanol, then stained with 0.1% crystal violet solution and counted in 3 random fields of view (200×). For wound healing assay, GC cells were seeded in six-well plates. A linear scratch wound was created by a 20 μl pipette tip in a confluent monolayer of cells. After 36 h of incubation in medium without FBS, the wound closure was observed and photographed under a microscope. The experiments were conducted in triplicate and repeated three times.

### Dual-luciferase reporter assay

We used two bioinformatic databases (miRcode and LncBase Predicted) to integrate the potential miRNA targets of LINC01939. The full-length lncRNA LINC01939 cDNA was cloned into a pmirGLO dual-luciferase Target Expression Vector (Promega, Madison, WI, USA) to construct the reporter vector pmirGLO-LINC01939-WT. To examine the binding specificity, the potential miR-17-5p binding sites were mutated which was named as pmirGLO-LINC01939-Mut. Dual-luciferase reporter assay was performed as described previously^37^. Briefly, HEK-293FT cells were co-transfected in 48-well plates with pmirGLO-LINC01939-WT or pmirGLO-LINC01939-Mut and miR-17-5p mimic or miR-control by Lipofectamine3000 (Life). At 48 h after transfection, luciferase activities of HEK-293FT cells were detected using the Dual-luciferase Reporter Assay System (Promega, Madison, WI, USA) according to the manufacturer’s protocol. The firefly luciferase activity was normalized by renilla luciferase activity. To confirm the direct interaction between miR-17-5p and EGR2, the full-length of EGR2 mRNA 3′-UTR and its mutant sequence were amplified using PCR and cloned into the pcDNA3.1 vector (Promega). 293FT cells were cultured into 6-well plates and co-transfected with 100 pmol of miR-17-5p mimic or miR-control with 2 ug of luciferase reporter plasmid and 200 ng of Renilla control reporter vector. Luciferase activity was measured 48 h post-transfection by the Dual-luciferase Reporter Assay System (Promega). The results were normalized to Renilla luciferase activity.

### In vivo metastasis assay

Female BABL/c athymic nude mice (4–5 weeks old) were purchased form the Shanghai Experimental Animal Center of Chinese Academic of Sciences (Shanghai, China). Two groups (8 mice in each group) were kept under pathogen-free conditions. For experimental metastasis assays, the SGC7901-LINC01939 and SGC7901-control cells

(2 × 10^6^ cells/mouse) were injected into the tail vein of nude mice. After 8 weeks, all mice were sacrificed. The metastatic nodes in the lung were counted by general observation and haematoxylin-eosin (H & E) staining.

### Western blot assay

Standard western blot assay was performed as previously stated^38^. In Brief, Total proteins were extracted from GC cells by RIPA cell lysis buffer. 30 μg of lysate proteins were separated using SDS-PAGE gels and then transferred onto polyvinylidene fluoridemembranes (PVDF, Millipore, USA). After incubating with primary antibodies overnight, the membranes were incubated with HRP-conjugated secondary antibody for 2 h with gentle shake. The antibodies against N-cadherin (1:1000), E- cadherin (1:1000), Vimentin (1:1000), α-catenin (1:1000), and GAPDH (1:5000) were purchased from Cell Signaling Technology (Cell Signaling Technology, USA). The antibodies against EGR2 (1:500) were purchased from Proteintech Group (Wuhan, China). Finally, The protein bands were visualized and captured by a Tanon detection system using the SuperSignal^®^ ECL Kit (Pierce, USA).

### Statistical analysis

The results presented as means ± standard deviation (SD) from at least three separate experiments. The Student *t*-test was used to compare the difference of means between two groups. The association of LINC01939 expression with clinicopathological parameters was evaluated by chi-square test or Fisher’s exact test. Kaplan-Meier curve and log-rank test were performed to estimate the predictive value of LINC01939 on outcome of GC patients. Spearman Pearson correlation analysis were conducted to assess the correlation between LINC01939 and miR-17-5p expression. *P* *<* 0.05 was considered to indicate a statistically significant difference.

## Supplementary information

Supplementary Table S1

Supplementary Table S2

Supplementary Figure S1

Supplementary Figure S2

Supplementary Figure S3

Supplemental figure legends
