# Supplementary material for: LINC01939 inhibits the metastasis of gastric cancer by acting as a molecular sponge of miR-17-5p to regulate EGR2 expression
Source: Cell Death Dis. 2019 Jan 25;10(2):70. doi: 10.1038/s41419-019-1344-4 (PMC6347617; doi:10.1038/s41419-019-1344-4)
Supplement: Supplementary file 1 — Supplementary Table S1 [file 41419_2019_1344_MOESM1_ESM.docx]

| **Supplementary Table S1**. **Correlation of LINC01939 expression with** **clinicopathological parameters in gastric cancer** | | | | |
| --- | --- | --- | --- | --- |
| Parameters | No of patients | LINC01939 Expression (%) | | *P*-value ^a^ |
|  |  | Low | High |  |
| Gender |  |  |  |  |
| Female | 63 | 30 (47.6%) | 33 (52.4%) | 0.540 |
| Male | 97 | 51 (52.6%) | 46 (47.4%) |  |
| Age (years) |  |  |  |  |
| < 60 | 89 | 47 (52.8%) | 42 (47.2%) | 0.536 |
| ≥ 60 | 71 | 34 (47.9%) | 37 (52.1%) |  |
| Tumor size (cm) |  |  |  |  |
| < 5 | 71 | 34 (47.9%) | 37 (52.1%) | 0.536 |
| ≥ 5 | 89 | 47 (52.8%) | 42 (47.2%) |  |
| Differentiation status |  |  |  |  |
| Well/Moderate | 28 | 17 (60.7%) | 11 (39.3%) | 0.240 |
| Poor | 132 | 64 (48.5%) | 68 (51.5%) |  |
| TNM stage |  |  |  |  |
| I-II | 55 | 17 (30.9%) | 38 (69.1%) | 0.000 |
| III-IV | 105 | 64 (61.0%) | 41 (39.0%) |  |
| Lymph node metastasis |  |  |  |  |
| No | 44 | 13 (29.5%) | 31 (70.5%) | 0.001 |
| Yes | 116 | 68 (58.6%) | 48 (41.4%) |  |
| Distant metastasis |  |  |  |  |
| No | 131 | 65 (49.6%) | 66 (50.4%) | 0.588 |
| Yes | 29 | 16 (55.2%) | 13 (44.8%) |  |
| Peritoneum dissemination |  |  |  |  |
| No | 143 | 71 (49.7%) | 72 (50.3%) | 0.474 |
| Yes | 17 | 10 (58.8%) | 7 (41.2%) |  |
| ^a^ *P* < 0.05, Chi-square test. TNM stage, tumor-node-metastasis stage. | | | | |
