# Supplementary material for: LINC01939 inhibits the metastasis of gastric cancer by acting as a molecular sponge of miR-17-5p to regulate EGR2 expression
Source: Cell Death Dis. 2019 Jan 25;10(2):70. doi: 10.1038/s41419-019-1344-4 (PMC6347617; doi:10.1038/s41419-019-1344-4)
Supplement: Supplementary file 2 — Supplementary Table S2 [file 41419_2019_1344_MOESM2_ESM.docx]

**Supplementary Table S2. The primer sequences of target genes used in this study**

| Name | Sequence 5'-3' |
| --- | --- |
| LINC01939-1-forward | CAAGAAGCAGAAGCTAAGGG |
| LINC01939-1-reverse | GGAACTATGTATGGCACCAG |
| LINC01939-2-forward | TTGCAGCAGTTGGATGTG |
| LINC01939-2-reverse | GGAACTATGTATGGCACCAG |
| has-miR-17-5p-forward | CGGCGGCAAAGTGCTTACAG |
| has-miR-17-5p-reverse | GTGCAGGGTCCGAGGT |
| pri-miR-17-29-forward | CATCTACTGCCCTAAGTGCTCCTT |
| pri-miR-17-29-reverse | GCTTGGCTTGAATTATTGGATGA |
| β-actin-forward | AATCGTGCGTGACATTAAGGAG |
| β-actin-reverse | ACTGTGTTGGCGTACAGGTCTT |
| small nuclear RNA(U6)-forward | CGCTAGCACATATCGGCTA |
| small nuclear RNA(U6)-reverse | TTCTGCGACGAATTTGTCAT |
| c-Myc-forward | GGAGGCTATTCTGCCCATTT |
| c-Myc-reverse | CGAGGTCATAGTTCCTTGTTGGT |
| TGFBR2-forward | GTAGCTCTGATGAGTGCAATGAC |
| TGFBR2-reverse | CAGATATGGCAACTCCCAGTG |
| PTEN-forward | TTTGAAGACCATAACCCACCAC |
| PTEN-reverse | ATTACACCAGTTCGTCCCTTTC |
| TIMP2-forward | GGAGGCTATTCTGCCCATTT |
| TIMP2-reverse | GGGGCCGTGTAGATAAACTCTAT |
| EGR2-1-forward | TCTTCCCAATGATCCCAGACT |
| EGR2-1-reverse | TTACGGATTGTAGAGAGTGGAGT |
| EGR2-2-forward | TCAACATTGACATGACTGGAGAG |
| EGR2-2-reverse | AGTGAAGGTCTGGTTTCTAGGT |
| GAPDH-forward | CTCCTCCTGTTCGACAGTCAGC |
| GAPDH-reverse | CCCAATACGACCAAATCCGTT |
| miR-215-forward | GGGTCCGAGGTATTCGCACT |
| miR-215-reverse | CGATGACC-TATGAATTGACAGACG |
| miR-422a-forward | GCATACCGCTATGCCTAATGGTG |
| miR-422a -reverse | GTGCAGGAGGTTCCGGT |

For confirming our results, we performed RT-PCR to investigate the relative expression of LINC01939 or EGR2 by two sets of specific primers for the two RNAs in this study.
