# Supplementary figures and images for: LINC01939 inhibits the metastasis of gastric cancer by acting as a molecular sponge of miR-17-5p to regulate EGR2 expression
Source: Cell Death Dis. 2019 Jan 25;10(2):70. doi: 10.1038/s41419-019-1344-4 (PMC6347617; doi:10.1038/s41419-019-1344-4)

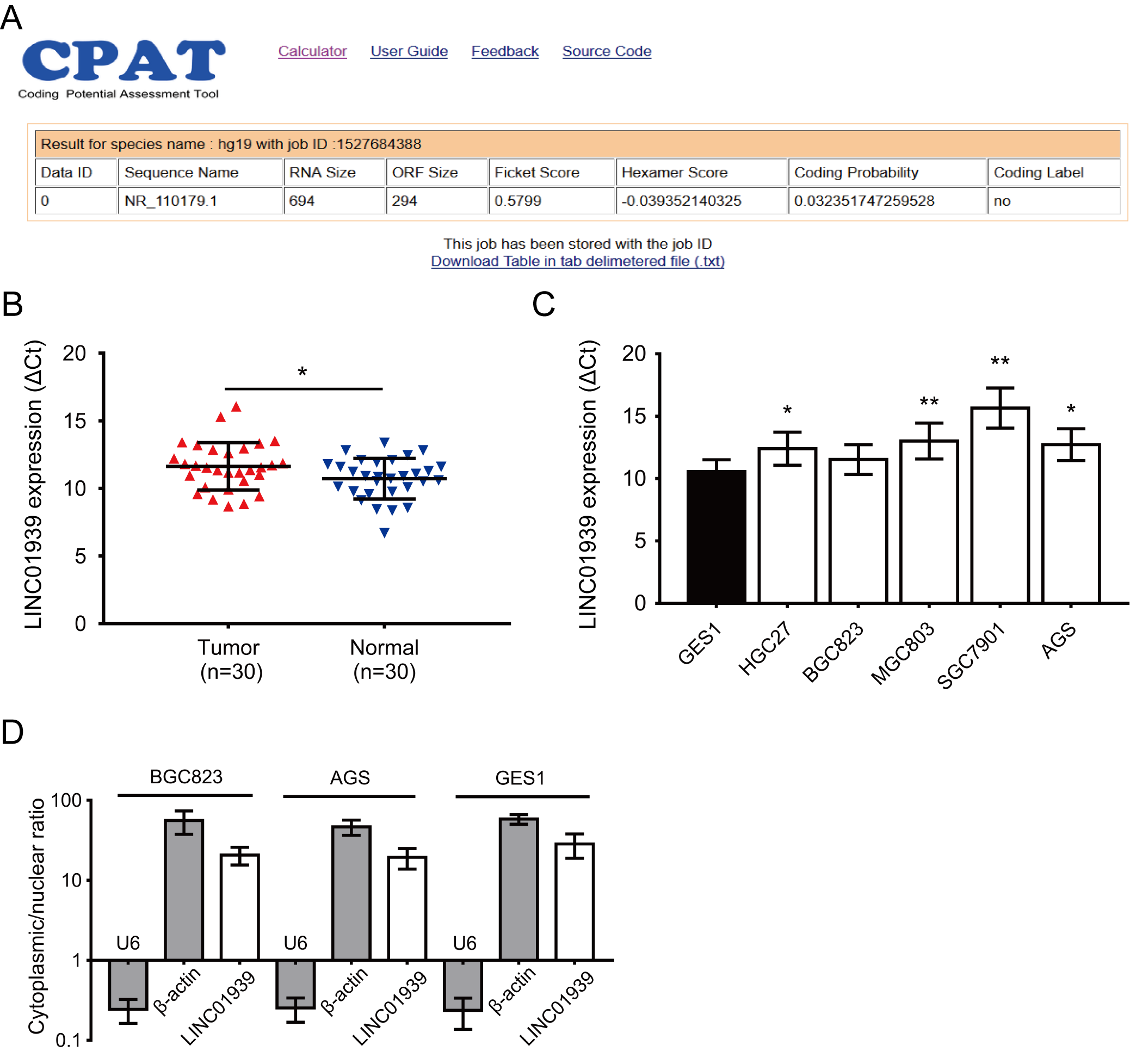

Supplement: Supplementary file 3 — Supplementary Figure S1 [file 41419_2019_1344_MOESM3_ESM.tif]

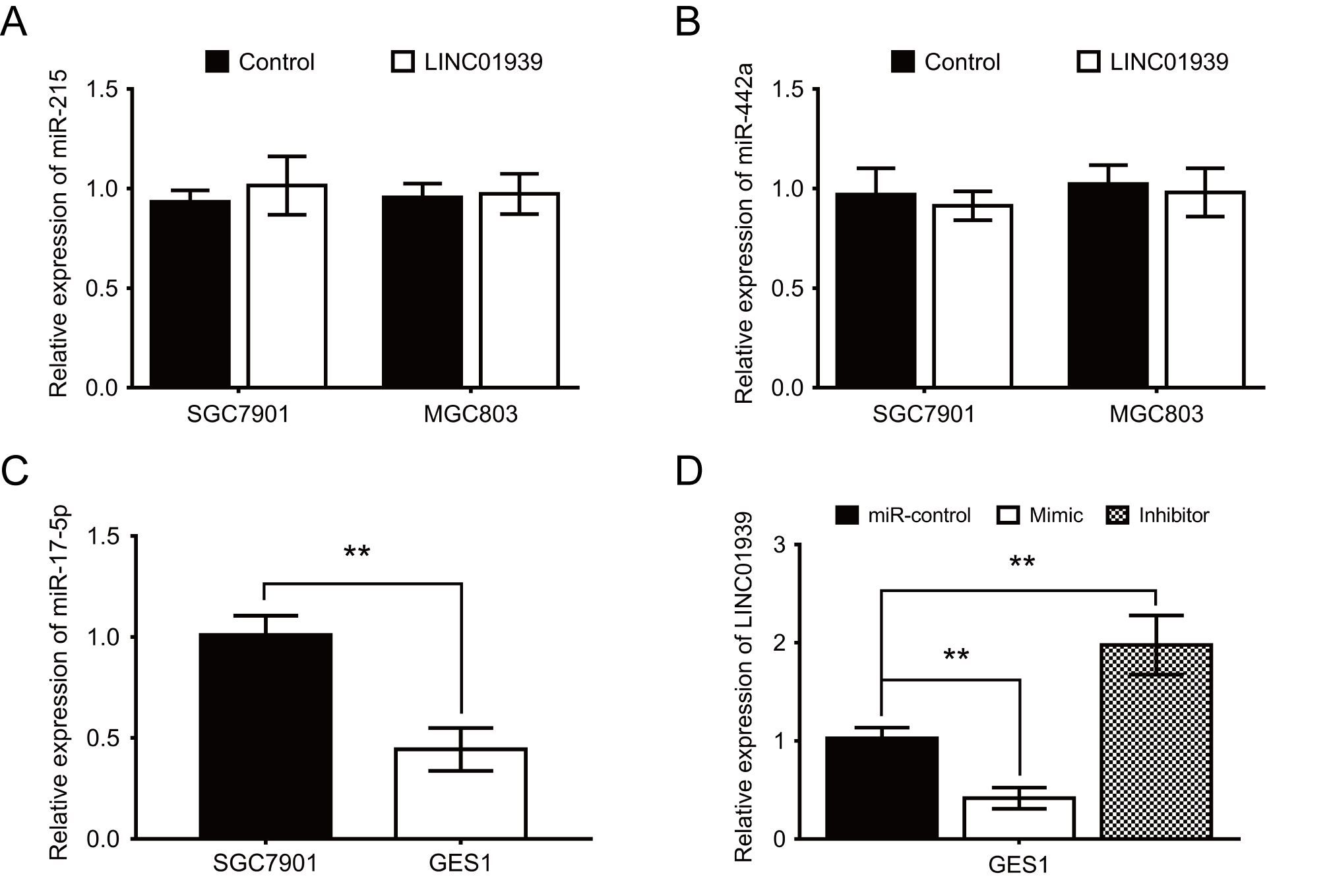

Supplement: Supplementary file 4 — Supplementary Figure S2 [file 41419_2019_1344_MOESM4_ESM.tif]

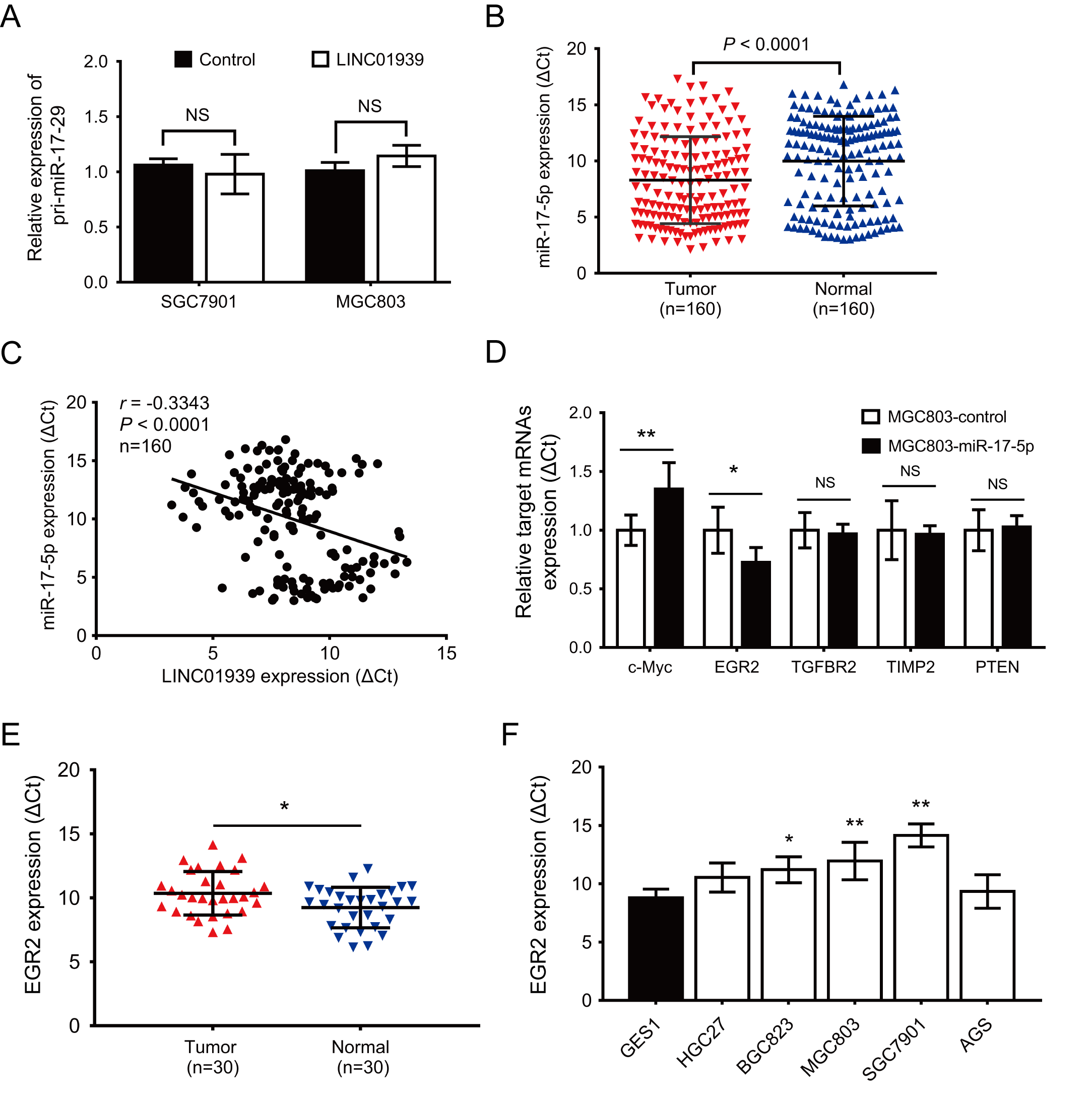

Supplement: Supplementary file 5 — Supplementary Figure S3 [file 41419_2019_1344_MOESM5_ESM.tif]
