# Supplementary material for: LINC01939 inhibits the metastasis of gastric cancer by acting as a molecular sponge of miR-17-5p to regulate EGR2 expression
Source: Cell Death Dis. 2019 Jan 25;10(2):70. doi: 10.1038/s41419-019-1344-4 (PMC6347617; doi:10.1038/s41419-019-1344-4)
Supplement: Supplementary file 6 — Supplemental figure legends [file 41419_2019_1344_MOESM6_ESM.docx]

**LINC01939 inhibits the metastasis of gastric cancer by acting as a molecular sponge of miR-17-5p to regulate EGR2 expression**

Mi Chen^1,7^, Li Fan^1,7^, Si-Min Zhang^2^, Yong Li^3^, Peng Chen^1^, Xin Peng^1^, Dong-Bo Liu^4^, Charlie Ma^5^, Wen-Jie Zhang^6^, Zhen-Wei Zou^*,1^, Pin-Dong Li^*,1^

**Supplementary figure legends**

**Supplementary Figure S1. (A)** Coding Potential Assessment Tool (CPAT) predicted that LINC01939 had no ability to code a protein (coding probability (CP) < 0.364). **(B)** Relative expression of LINC01939 in 30 paired GC cancer tissues and matched normal tissues by RT-PCR assay using another set of specific primers for LINC01939. **(C)** RT-PCR assay further confirmed the expression levels of LINC01939 in a panel of GC cell lines using another set of specific primers for LINC01939. **(D)** RT-PCR analysis of LINC01939 in nuclear and cytoplasmic fractions of BGC823, AGS and GES1 cells. U6 and β-actin acted as nucleus and cytoplasm marker respectively (n=3). Error bars: mean ± SD. n=3, ^*^ *P* <0.05, ^**^*P* < 0.01.

**Supplementary Figure S2. (A) and (B)** Relative expression of miR-215 and miR-442a in SGC7901 and MGC803 cells with LINC01939 overexpression by RT-PCR assay. **(C)** Expression of miR-17-5p detected by RT-PCR in SGC7901 and GES1 cells. **(D)** Expression of LINC01939 after transfection of miR-17-5p mimic or inhibitor in GES1 cells. Error bars: mean ± SD. n=3, ^**^*P* < 0.01.

**Supplementary Figure S3. (A)** Effect of overexpression of LINC01939 on pri-miR-17-29 in SGC7901 and MGC803 cells. **(B)** Expression of miR-17-5p measured by RT-PCR assay in 160 paired GC tissues and matched normal tissues. Results were presented as Δcycle threshold (ΔCt) in tumor tissues relative to normal tissues. **(C)** Correlation between LINC01939 and miR-17-5p expression in 160 matched normal tissues. **(D)** Expression of five candidate mRNAs of miR-17-5p targeted genes confirmed by RT-PCR in MGC803-control and MGC803-miR-17-5p cells. **(E)** Relative expression of EGR2 in 30 paired GC cancer tissues and matched normal tissues by RT-PCR assay using another specific primer of EGR2. **(F)** RT-PCR assay further confirmed the expression levels of EGR2 in a panel of GC cell lines using another specific primer of EGR2. Error bars: mean ± SD. n=3, ^*^ *P* <0.05, ^**^*P* < 0.01, NS, no significant.
